# Sustained Reduction in Time to Data Entry in the Cystic Fibrosis Foundation Registry

**DOI:** 10.1097/pq9.0000000000000529

**Published:** 2022-01-21

**Authors:** Laura Nay, Jame’ Vajda, Sharon McNamara, Thida Ong

**Affiliations:** From the * Snohomish Health District, Seattle, Wash.; † Seattle Children’s Hospital, Seattle, Wash.; ‡ Division of Pulmonary and Sleep Medicine, Department of Pediatrics, University of Washington. Seattle, Wash.

## Abstract

**Introduction:**

Timely data entry into patient registries is foundational to learning health systems such as the Cystic Fibrosis Learning Network. The US Cystic Fibrosis Foundation Patient Registry (CFFPR) is an established registry that collects encounter data for clinical and research activities. Coordinators manually enter approximately 1,500 encounters annually at our institution, but there is limited evidence for interventions facilitating timely data entry. Our institution aimed to reduce the number of days between a clinical encounter and data entry into the CFFPR from an average of 43 days (range 0 to 183 days) to less than 30 days in a 3-month interval.

**Methods:**

Data coordinators tested interventions to address barriers in four themes: accountability, work burden, communication, and visibility using plan-do-study-act cycles. We used statistical process control charts to assess progress on average time of entry. Coordinators provided feedback about acceptability and satisfaction for process changes.

**Results:**

Initial interventions standardized process and reduced average time to data entry from 42.6 to 22.5 days in 3 months, but this process was not stable in the subsequent 6 months. Subsequent changes to increase metric visibility and improve team communication increased stability and decreased the average time to data entry to 23.0 days. Coordinators reported high satisfaction with process changes and have sustained improved time for over 2 years.

**Conclusions:**

This quality improvement project reduced and maintained data entry time by addressing significant barriers without additional personnel. Increased access to near real-time data in CFFPR accelerates learning for clinical care, quality improvement, and research.

## INTRODUCTION

Multiple collaborative networks organize as learning health systems to use data review and visible metrics to drive quality improvement (QI) and innovations.^1^ Accurate and timely data inputs are foundational to the structure of learning health systems and may require new or enhanced existing patient registries.^2^

In cystic fibrosis (CF), patient registries have been a longstanding and influential resource to advance care and clinical outcomes.^3^ Patient data registries are operational in over 20 countries, representing more than 72,000 individuals with CF worldwide.^3,4^ The US CF Foundation Patient Registry (CFFPR) collects over 350 patient-specific, encounter-based variables longitudinally^5,6^ and is supported by data coordinators from CF Foundation (CFF)-accredited care programs in the national care center network.

The CFFPR is used widely by clinicians and researchers. Both clinicians and researchers have identified the importance of technological infrastructure to support data entry as a tool in a learning health system model.^7^ In its current form, data input into CFFPR requires manual and time-intensive entry into an online data portal, known as PortCF. Timely data entry expands opportunities to use CFFPR to trend patient-specific clinical data, assess QI initiatives, and identify eligible patients for clinical research. Despite these multiple important uses for faster access to registry data, processes to facilitate timely data entry into CFFPR have not been widely disseminated.

In October 2016, CFF sponsored the formation of the CF Learning Network (CFLN) among 13 initial sites.^8^ The CFLN, in partnership with CFFPR, is structured as a learning health system to align data visibility and QI activities across participating programs.^1,9^ To establish timely data visibility across sites, the CFLN recommended programs input data into the CFFPR within 30 days of each clinical encounter, including clinic visits, laboratory, and pulmonary function only visits, and hospitalizations.

As a site in the first CFLN cohort, the Seattle Children’s Hospital (SCH) CF center identified variable timing for data entry into the CFFPR ranging from 0 to 6 months from the date of the encounter. Standard practice by data coordinators before the improvement project was to enter data as time permitted. The QI team aimed to reduce the average number of days between encounter and date of data entry from 43 to less than 30 days in 3 months. The purpose of this report is to describe the approach, themes, and interventions to achieve sustained and reliable, timely data entry into a patient registry.

## METHODS

This report is presented according to the Standards for Quality Improvement Reporting Excellence (SQUIRE 2.0) guidelines.^10^ This was a QI project that followed guidance from the SCH Institutional Review Board and did not require approval.

### Context/Setting

The SCH CF Center is a moderate-sized pediatric center providing care for approximately 200 people with CF, ranging from newborn to 21 years of age. Two full-time data coordinators supported through the CFF-funded center grant share responsibility for CFFPR data input. The coordinators are each supported by 0.05 Full-Time Equivalents (FTEs) for data entry, and the remainder of their time supports additional clinical and research activities. An average of 125 encounters, including clinic visits and hospitalizations, are entered into the CFFPR monthly. FTE support and the number of clinical encounters entered into CFFPR remained steady over the project period. Parental permission for minors is obtained for data entry into the CFFPR, with assent at 14 years of age and consent at 18 years of age. CFFPR publishes a manual for data entry guidance and has an annual deadline to enter any encounter from a calendar year, typically in the first quarter of the subsequent year.

The CF QI team consists of physicians, nurses, two parent partners, a clinic coordinator, QI coordinator, and a respiratory therapist that meets weekly; clinical and research staff join specific initiatives as relevant. A CFFPR data subgroup consisted of a research nurse manager who led the initiative, two CFFPR data coordinators, and a QI coordinator.

During the initial years in the CFLN, CFFPR provided monthly reports to each participating program displaying site-specific and network-wide measures, including the proportion of clinic encounters entered within 30 days. Our QI team also participated in monthly CFLN webinars and biannual community conferences with other CFLN sites to present activities and participate in QI educational workshops.

### Intervention Design

**Fig. 1.**
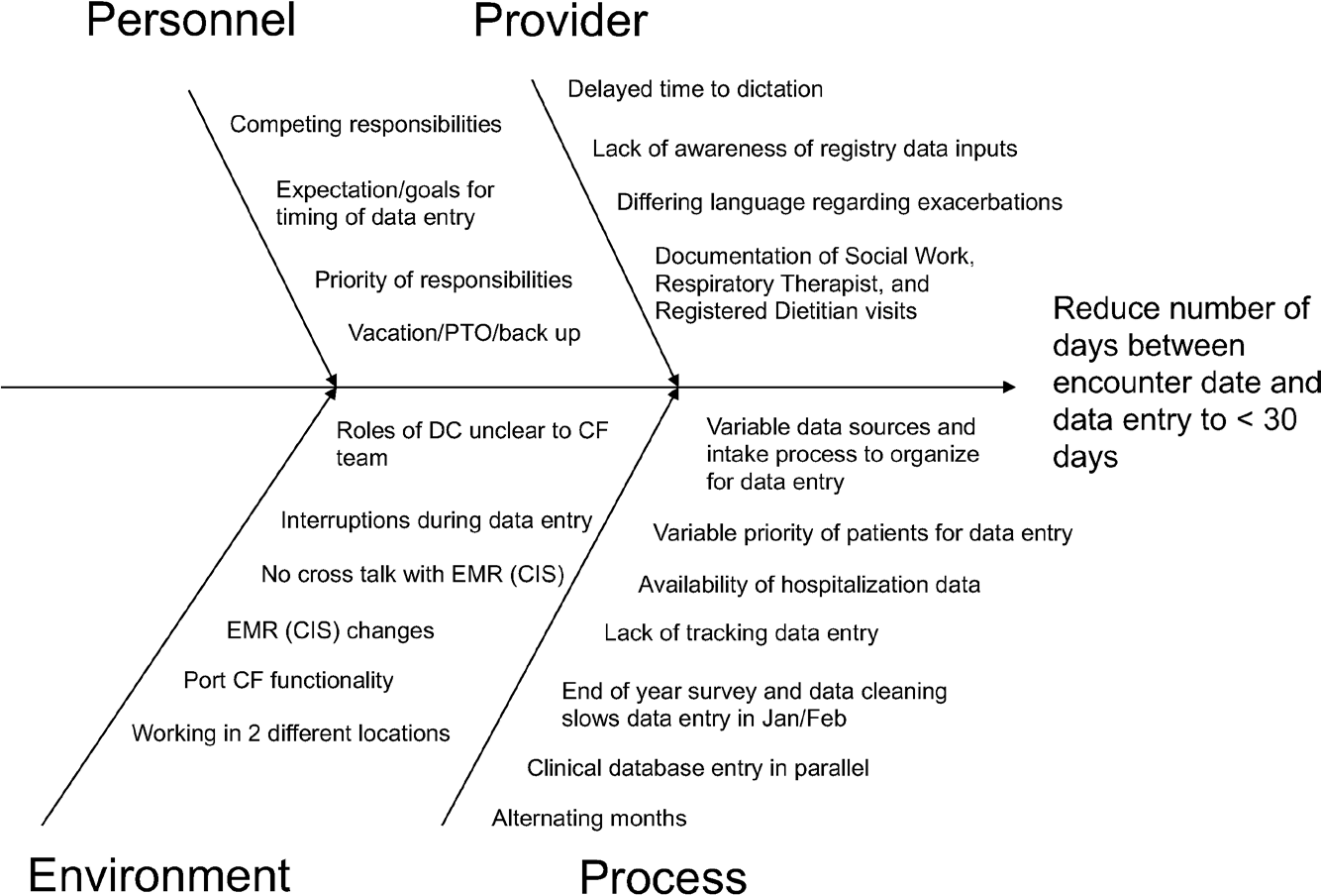
Cause and effect diagram organizing barriers and possible targets of action for timely data entry into the Cystic Fibrosis Foundation Patient Registry. EMR, electronic medical record.

To understand sources of barriers to timely data entry within current processes, the data subgroup team studied time, interruptions, and workload burden. The QI team organized barriers in a cause-effect diagram and determined action targets (Fig. 1).^11^ We studied process changes in rapid Plan-Do-Study-Act (PDSA) cycles.

To redesign data entry processes over the project period, the QI team prioritized interventions to improve barriers attributed to two themes: (1) accountability, defined as setting goals and attributing responsibilities and (2) work burden, defined as being aware of the distribution of work (Table 1). During the first round of PDSAs between October and December 2016, clear expectations of timely entry were agreed upon to increase accountability. Before the improvement project, the entered encounters were divided by month. Coordinators shared time with research projects or administrative duties in addition to data entry. Data entry time was protected and flexible across concurrent projects, however, entry on the monthly system created a periodic accumulation of data entry needs, overburdening the data coordinators. In the new process, the workload was rearranged in a biweekly (every other week) distribution. With this new method, the coordinators entered data more consistently and closer to the encounter in approximately 4 hours/week. We projected clinic and hospitalization assignments quarterly to allow data coordinators to plan proactively. The data entry workload was constant and easily balanced with other job responsibilities by limiting the accumulated hours needed to enter over a month. Progress was reported to the CF QI team at quarterly to semiannual intervals and the CFLN community at annual conferences.

**Table 1.** Summary of Interventions

Barrier Addressed	Intervention
**Theme 1: Accountability**	
No defined expectations for data entry timeliness	Data coordinators and QI team set goal to decrease average days to data entry to less than 30 d
**Theme 2: Work Burden**	
Bolus data entry at the end of each month	Data entry assignments changed from monthly to 2-wk intervals
Difficulty planning data entry around competing responsibilities	Preassigned clinic entry dates on a quarterly basis with equitable distribution to allow data coordinators to plan proactively
**Theme 3: Visibility**	
Lack of visible timeliness metric	Queried PortCF on a biweekly basis to assess data timeliness. Results communicated with data coordinators and research nurse manager
Data coordinators unaware of inpatient hospitalizations until after patient discharge	Updated hospitalization tracking spreadsheet daily with inpatient admission dates and weekly with inpatient discharge dates
**Theme 4: Communication**	
Lack of communication regarding data coordinator workload	Implemented shared spreadsheet tracking number of clinic encounters, date of CFFPR entry, and number of encounters entered
	Held CFFPR data subgroup meetings every other week to review data timeliness, address special causes of data entry interruptions, and adjust workloads to reflect competing projects
	Established weekly dedicated time for data entry and communicated this to the CF team to minimize interruptions

Improvements made after the first round of PDSAs could not maintain a stable process. Issues reported by data coordinators included: interrupted workflow with competing priorities such as clinic coordination and research database management, lapses in electronic medical record availability, unclear communication between coordinators and team, limited personnel due to an unexpected leave of absence, and lack of performance visibility. In addition, initial process changes targeted input of clinic encounters and did not include hospitalization encounters. Review of hospitalization encounters found that delayed entry was due to a protracted notification process for coordinators to learn about patient admission and discharge dates. Updated barriers fell under two themes: (1) to increase visibility and (2) to improve communication (Table 1). The second round of PDSA cycles between June and November 2017 targeted these barriers (Table 1). We improved visibility by adopting a process for data coordinators to review admissions to hospitalizations daily. The QI coordinator also developed biweekly charts of progress to outcomes, and the data subgroup team reviewed these charts at meetings every other week. Communication improved between coordinators by implementing a shared spreadsheet between data coordinators to track data entry assignments. Concerns about competing priorities were discussed at biweekly data subgroup team meetings and were addressed by increasing the flexibility of data entry assignments as needed between the two coordinators. Data coordinators scheduled weekly dedicated day and time for data entry and communicated expectations to the CF team to minimize work interruptions. The hospital-wide lapse in electronic medical record availability and leave of absence were considered out of scope and not addressed during this project.

### Study of the Interventions/Measures/Analysis

The primary outcome was the average number of days between the date of clinical encounter and the date of data entry. We queried monthly data from PortCF, the portal interface with the CFFPR and all clinical encounters (clinic visits and hospitalizations) were included in the analysis.

Process measures estimated feasibility of the changes, including daily time recorded by data coordinators to enter data and use a shared spreadsheet to track the encounter numbers entered out of the total clinical encounters for assigned clinics weekly.

The team used statistical process control charts to assess if the interventions improved variation in the process of data entry. Specifically, we created average (X-bar) and SD (S) charts for continuous data.^12^ Initial control limits were derived from PortCF data for 14 months before joining the CFLN (August 2015 through September 2016). Special cause variation was defined if points were outside the control limits (>3 SDs from the mean) and if remained stable following PDSA cycles, we adjusted upper and lower control limits.

Competing workload, priorities, and interruptions in workflow, such as a disruption in the electronic medical record availability, were recorded in real-time by the data coordinators during review of special cause variation in statistical process control charts. Data were regularly shared with the CF QI team and the CF Center Director. Data coordinators provided ongoing input to the larger CF QI team with observations about process changes and satisfaction. Data coordinator job satisfaction was measured by recording qualitative statements shared during regular QI and data subgroup team meetings.

## RESULTS

### Decrease in Average Time to Data Entry

**Fig. 2.**
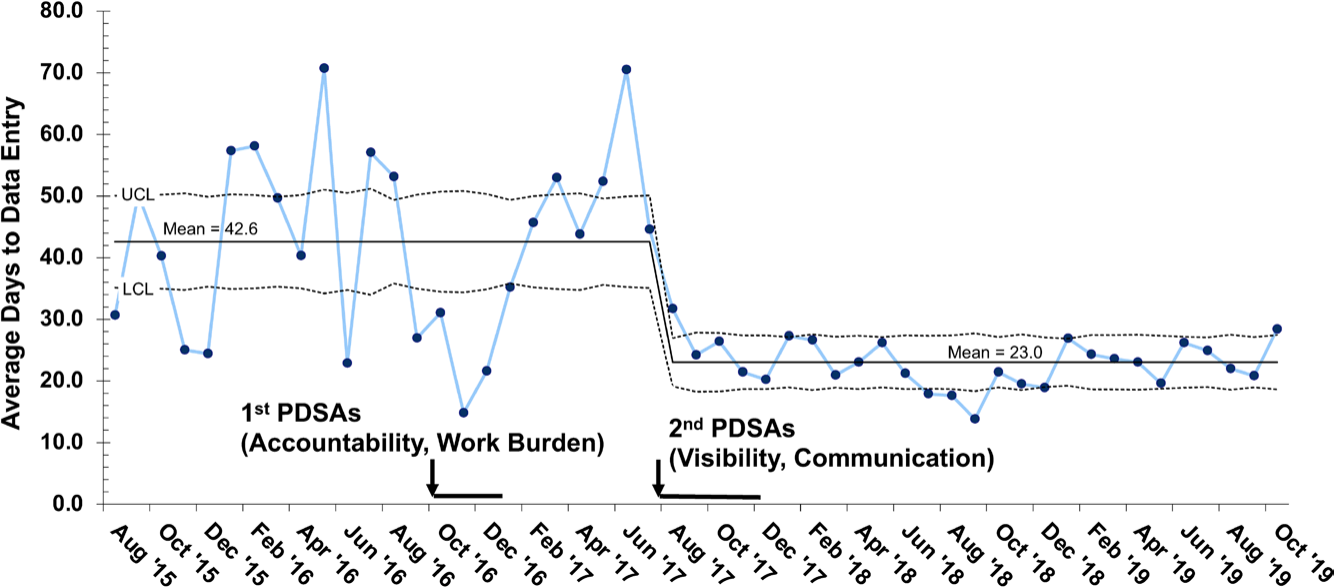
X-bar control chart of average days to data entry in the Cystic Fibrosis Foundation Patient Registry. LCL, lower control limit; PDSAs, plan-do-study-act cycles; UCL, upper control limit.

**Fig. 3.**
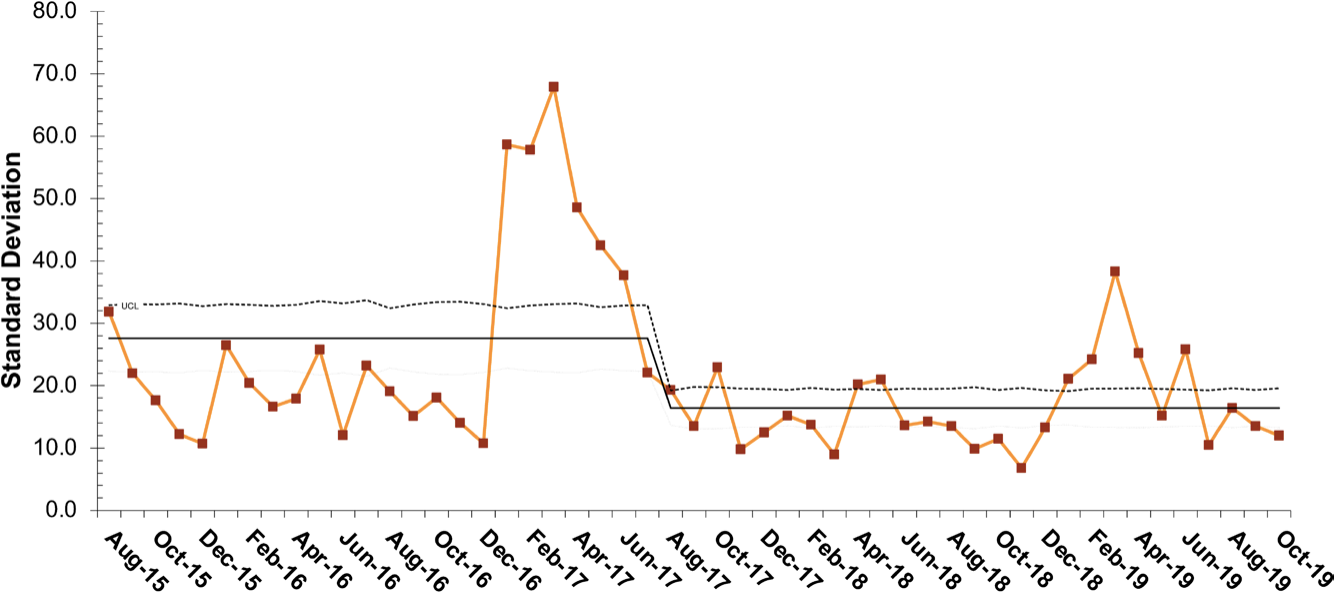
SD (S)-control chart per month of average days to data entry in the Cystic Fibrosis Foundation Patient Registry. LCL, lower control limit; UCL, upper control limit.

Rapid reduction in the average time from clinic encounter to PortCF data entry was seen with PDSA cycles over the first 3 months, decreasing mean time from 42.6 to 22.5 days at goal (Fig. 2). We identified special cause with the PDSA cycles; however, we were not able to sustain a stable process (Fig. 2). Data entry time was variable with an average increased to 50.3 days over the subsequent 6 months. Following the second round of PDSAs, the average data entry time decreased to 23.0 days. The standard deviations of the monthly measurements also significantly decreased as noted in the S chart (Fig. 3). These improvements have been sustained for over two years.

### Impact on CF Center

Data coordinators reported positive impacts, including a more manageable work structure that decreased daily stress and overwork, added collaboration across the QI and clinic teams, and increased overall job satisfaction. Data coordinators felt a sense of accomplishment by achieving set goals and received recognition from peers and job performance reviews. Coordinators reported limited negative comments but recalled concerns at the start of the project that they may be scrutinized for poor performance if goals were not achieved. Additional comments included concerns for added work needed to develop processes to support the organization of data entry. Data coordinators noted that although completing a shared spreadsheet and reviewing data added work upfront, it allowed for improved communication between the data coordinators that were adapted through PDSA cycles. Both data coordinators felt the experience of the improvement project, and the change in processes led to greater job satisfaction and joy in work.

Additional CF clinical team members also reported positive reactions and changed behaviors in response to this QI project. CF physicians noted an increase in available CFFPR data for review and subsequently felt accountable to improve timeliness in completing their entries into the medical record. This led to an upstream change to support available data for timely entry. The early success of this initiative empowered the CF QI team to take on additional QI projects and increased our center’s use of locally entered CFFPR data as metrics for outcomes improvement. The CF QI team also shared successes and challenges with other sites in the CFLN and participated in creating a collection of change ideas to facilitate CFFPR data entry across the learning network.

## DISCUSSION

Our CF center successfully decreased time to CFFPR data entry through process redesign and without additional staff. Setting goals (targeting accountability) and distributing data entry tasks (reducing the work burden) were important foundations for improvement efforts but insufficient to maintain timely entry. Data coordinators embedded changes into the daily workflow to augment communication and visibility of timely data measures. This project achieved a sustained improvement effect to reach data entry within 30 days from the clinical encounter for over two years.

The CFFPR is an exemplary encounter-based disease registry, representing over 80% of people with CF in the United States.^5^ Over 200 clinics contribute to the CFFPR, and clinic staff enter the data into a web-based portal through five electronic data capture forms.^5^ Although contribution to the CFFPR is mandatory for CFF-accredited centers, clinics self-organize to determine their own allocation of resources and supports to enter this data in a timely fashion. The CFLN as a subset of improvement-minded teams from the massive care center network offers a unique opportunity to identify, share, and unify timely data practices into the CFFPR. Clinics in mature learning networks, such as ImproveCareNow have also encountered the burden of cumbersome data entry processes preventing sites from both participation and contribution.^13^ Technology-based strategies such as direct upload from electronic medical records are important data features to streamline this work, however, can be burdened by various system requirements and information technology expertise.^13^ Pharmacies that have focused on timely data entry to immunization registries have also pointed to the need for concrete technological changes to enhance entry.^14^ We anticipate and report strategies for improvement teams to parallel ongoing technological advances to registry data entry processes. Registry team improvement methods coupled to existing and evolving technological strategies are an anticipated powerful combination to facilitate data sharing and further enhance registry functions.

Critical components of the adopted changes in this study were early design and ongoing input from the data coordinators, that is, the frontline team members doing the work. Strong leadership defined aims and partnership with the QI team and data coordinators carried through strategies to maintain success. Achieving goals increased job satisfaction and engagement across the CF team. The promotion of meaningful work across a multidisciplinary team is a fundamental contributor to increased productivity and efficiency.^15^ This team dynamic is congruent with a conceptual model for high-performance work systems to empower the frontline staff, align leadership, and engage staff to improve outcomes.^16^

The impact of timely data entry can be appreciated on additional fronts. Decreased average time to data entry across multiple sites was a major success of the pilot phase of the CFLN. The CFLN has positioned itself to have multiple programs regularly using real-time data to investigate population health outcomes (eg, acute drops in lung function).^17^ The CFFPR has concurrently expanded functionality through CFSmartReports, a web-based reporting tool for clinical point-of-care information.^18^ Shared registry information is a cornerstone for learning networks to share knowledge seamlessly and evaluate the impact of planned interventions.^19^

CF programs in the CFF Care Center Network are also well-poised to improve and benefit from timely data entry processes. Across the globe, CF programs rely on registry data to target outcomes improvement.^20–23^ Multiple programs report active use of the CFFPR to define targets and metrics for QI.^24^ Furthermore, training in QI methods and team-based QI work are widespread in CF teams, as seen through learning and leadership collaborative opportunities.^25,26^ Given the existing QI capacity of teams, improvement ideas for timely data entry into CFFPR are likely to spread across the larger CFF Care Center Network.

We share our local experiences with challenges to maintain a stable and reliable process for timely data entry. In doing so, we hope other teams approaching these improvements may consider similar themes for accountability, work burden, visibility, and communication. Future directions for this work include refining timely data practices across multiple clinics in the CFLN and spread throughout the CFF Care Center Network. Comparisons of timely data entry among CFF Care Centers and qualitative analysis may also reveal important benchmarking practices and insights for timely data practices and resources and FTE support for this work. We also advocate for institutions to support the productivity of local QI teams to maintain these foundational processes for data management and project coordination. As seen in other frameworks for sustainable QI initiatives, leadership, oversight, and funding support are integral to drive acceleration of improved outcomes.^27,28^

There are some limitations to our work. First, we did not quantitatively track balancing measures such as time taken away from competing priorities. Given the already added burden of tracking data entry, we report positive and negative aspects of the process changes from comments. Second, QI teams have their own resource limitations, to which our specific changes may not be applicable. Third, the themes and framework that contributed to this project’s success may not be generalizable to other institutions but are reported here so others can modulate or amplify our interventions to achieve success.

In summary, this QI project improved and sustained the timeliness of data entry into the CFFPR and created overarching team-based engagement through easily adoptable interventions without extra personnel. QI initiatives to improve the timeliness of data entry into CFFPR are warranted to accelerate learning health systems and contribute to the use of point-of-care data to inform clinical care improvements and research opportunities.

## ACKNOWLEDGMENTS

We would like to acknowledge past and present members of the SCH CF QI Team. Special thanks to Marcella Blackledge, CF Data Coordinator, Amanda Lu, and Dr. Ronald Gibson for their support in this project. We also thank the members and teams of the CF Learning Network.

Preliminary data presented at the 31st Annual North American Cystic Fibrosis Conference, November 2–4, 2017, Indianapolis, Indiana.

## DISCLOSURE

Dr Ong has received honoraria from Cincinnati Children’s Hospital Medical Center to serve on the Cystic Fibrosis Foundation CF Learning Network Leadership Team. The other authors have no financial interest to declare in relation to the content of this article.
